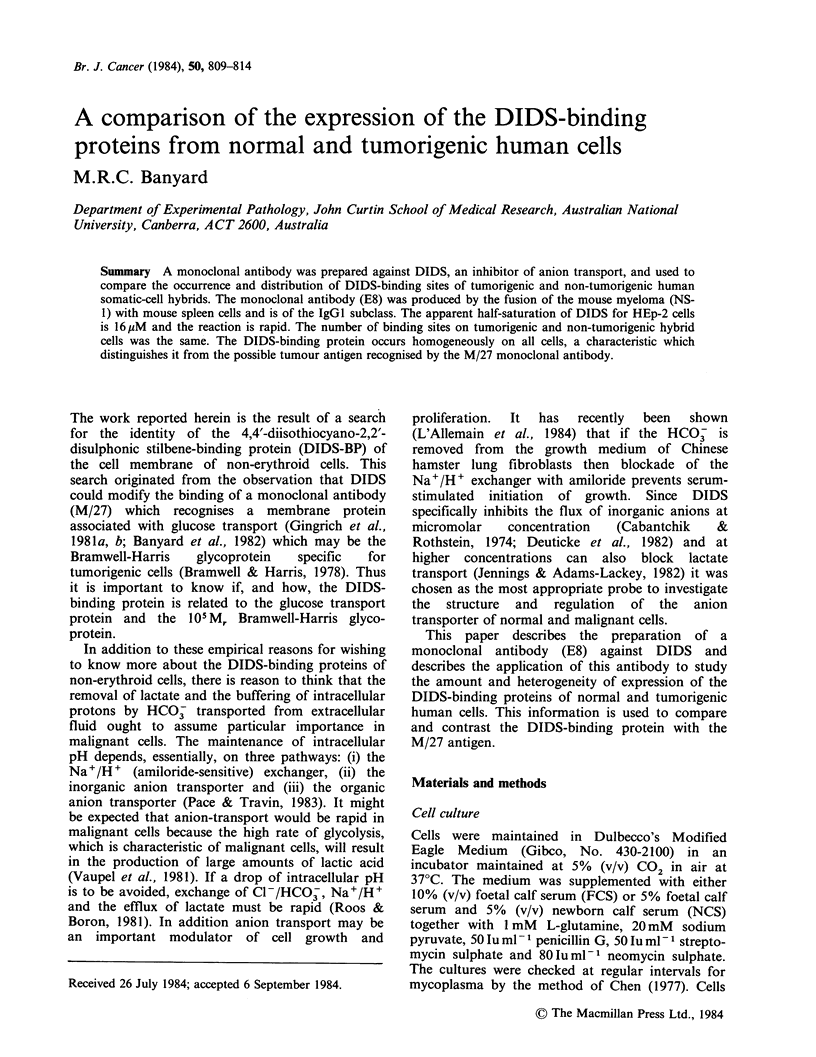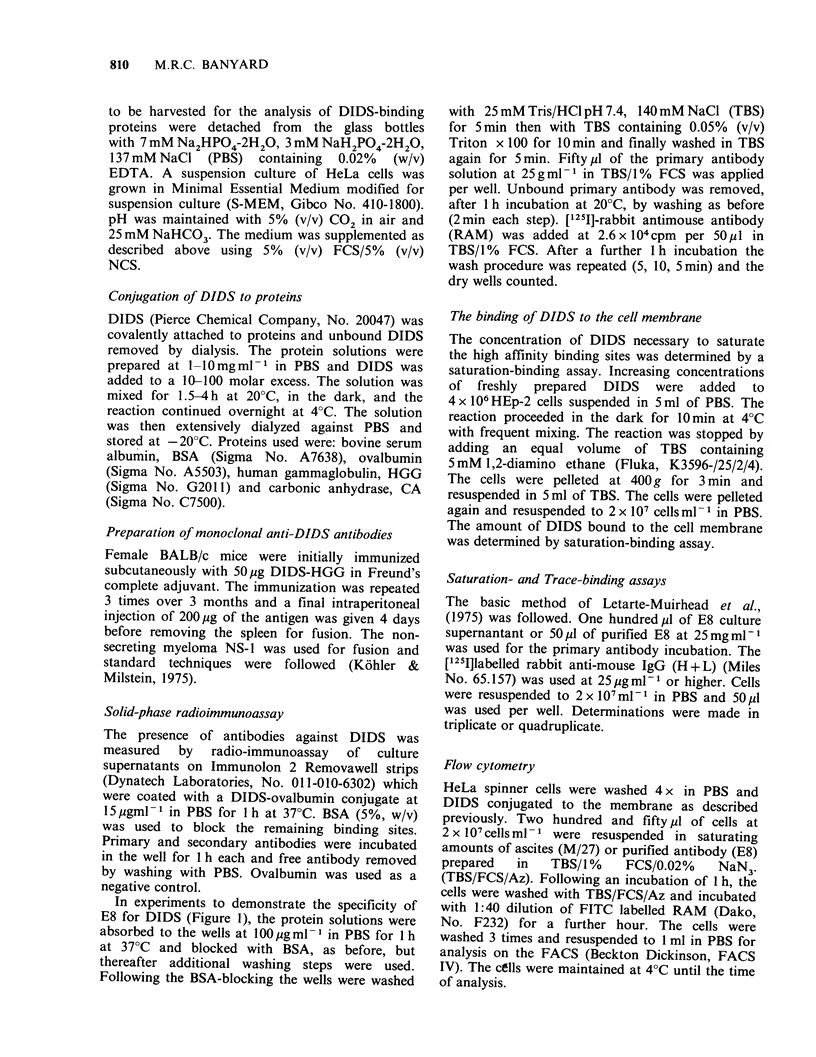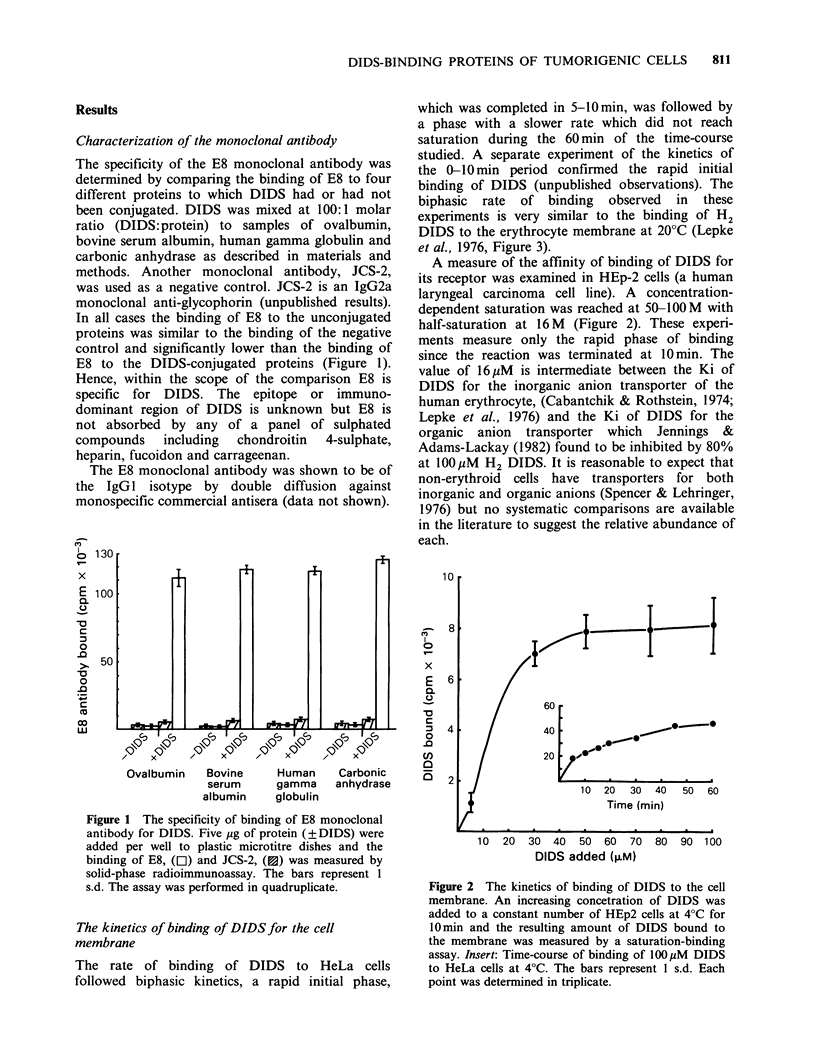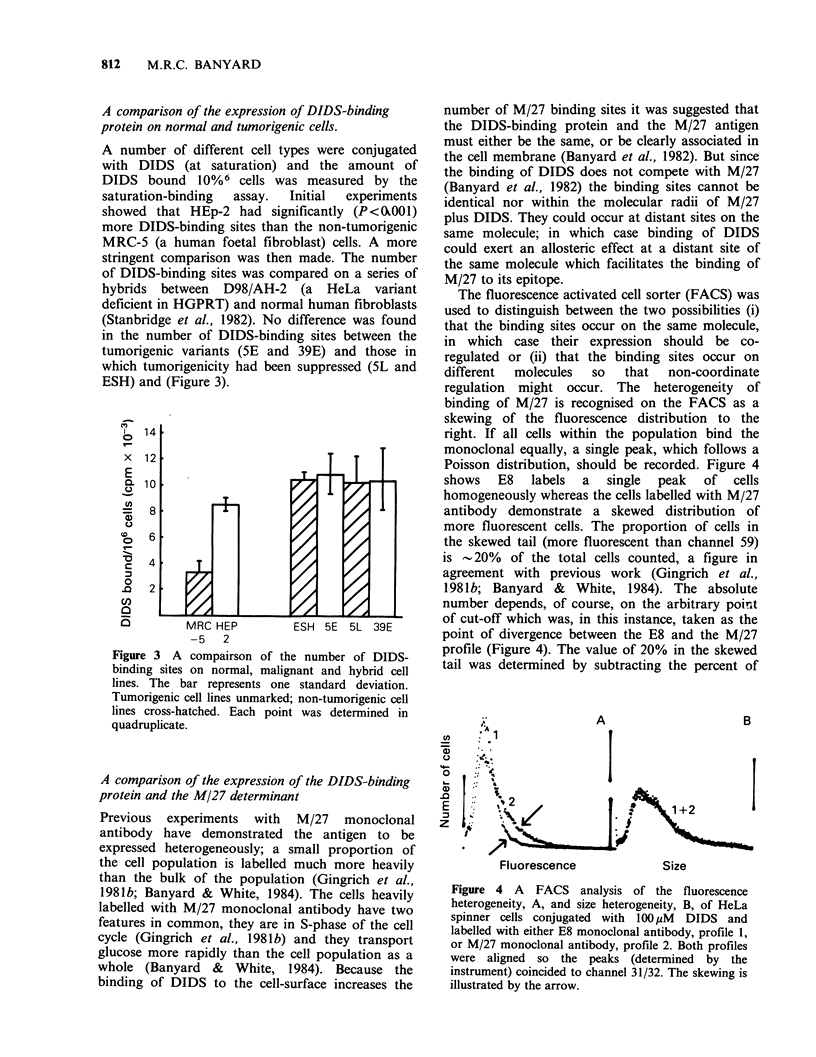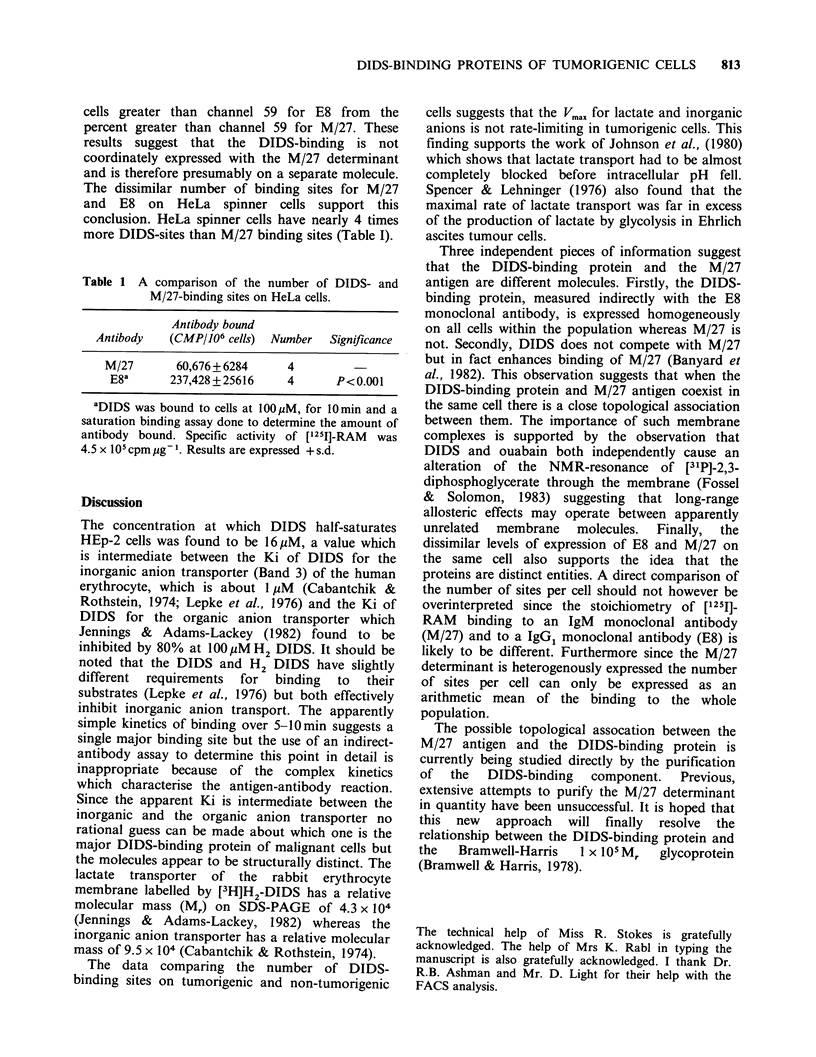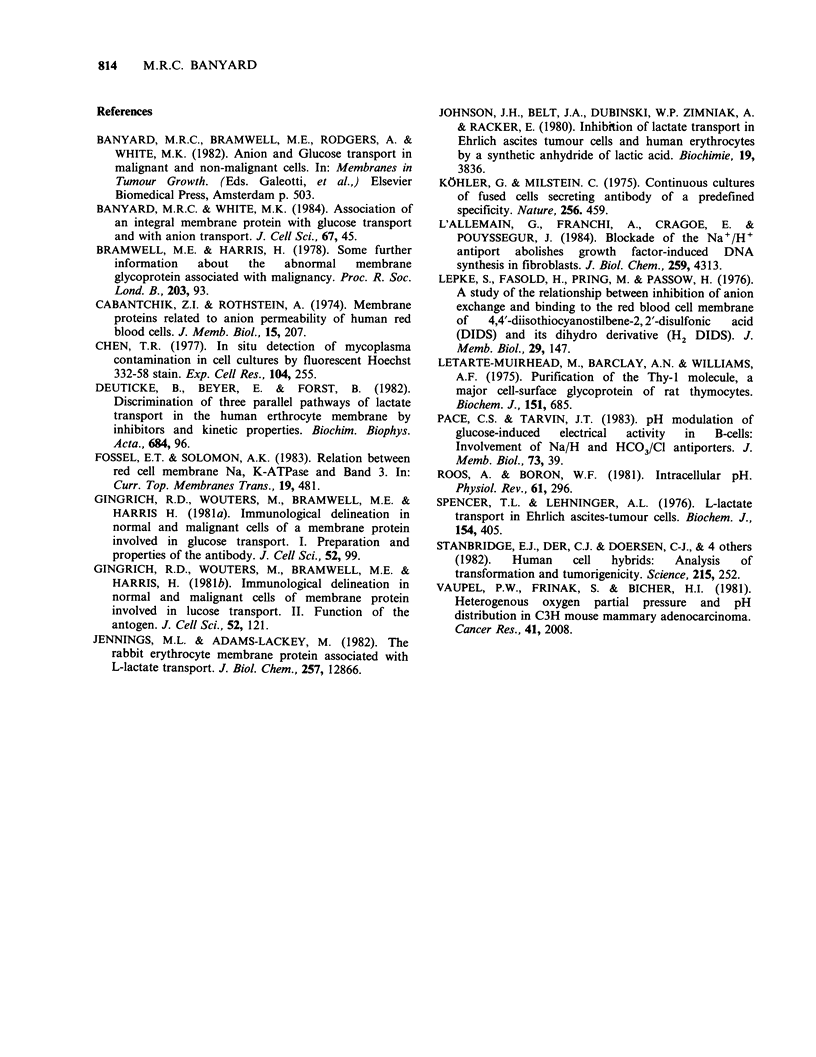# A comparison of the expression of the DIDS-binding proteins from normal and tumorigenic human cells.

**DOI:** 10.1038/bjc.1984.260

**Published:** 1984-12

**Authors:** M. R. Banyard

## Abstract

A monoclonal antibody was prepared against DIDS, an inhibitor of anion transport, and used to compare the occurrence and distribution of DIDS-binding sites of tumorigenic and non-tumorigenic human somatic-cell hybrids. The monoclonal antibody (E8) was produced by the fusion of the mouse myeloma (NS-1) with mouse spleen cells and is of the IgG1 subclass. The apparent half-saturation of DIDS for HEp-2 cells is 16 microM and the reaction is rapid. The number of binding sites on tumorigenic and non-tumorigenic hybrid cells was the same. The DIDS-binding protein occurs homogeneously on all cells, a characteristic which distinguishes it from the possible tumour antigen recognised by the M/27 monoclonal antibody.


*Br. J. Cancer* (1984), **50**, 809–814

### A comparison of the expression of the DIDS-binding proteins from normal and tumorigenic human cells

M.R.C. Banyard

*Department of Experimental Pathology, John Curtin School of Medical Research, Australian National University, Canberra, ACT 2600, Australia*

**Summary** A monoclonal antibody was prepared against DIDS, an inhibitor of anion transport, and used to compare the occurrence and distribution of DIDS-binding sites of tumorigenic and non-tumorigenic human somatic-cell hybrids. The monoclonal antibody (E8) was produced by the fusion of the mouse myeloma (NS-1) with mouse spleen cells and is of the IgG1 subclass. The apparent half-saturation of DIDS for HEp-2 cells is 16 $\mu$M and the reaction is rapid. The number of binding sites on tumorigenic and non-tumorigenic hybrid cells was the same. The DIDS-binding protein occurs homogeneously on all cells, a characteristic which distinguishes it from the possible tumour antigen recognised by the M/27 monoclonal antibody.

The work reported herein is the result of a search for the identity of the 4,4′-diisothiocyano-2,2′-disulphonic stilbene-binding protein (DIDS-BP) of the cell membrane of non-erythroid cells. This search originated from the observation that DIDS could modify the binding of a monoclonal antibody (M/27) which recognises a membrane protein associated with glucose transport (Gingrich *et al.*, 1981*a*, *b*; Banyard *et al.*, 1982) which may be the Bramwell-Harris glycoprotein specific for tumorigenic cells (Bramwell & Harris, 1978). Thus it is important to know if, and how, the DIDS-binding protein is related to the glucose transport protein and the $10^5 M_r$ Bramwell-Harris glycoprotein.

In addition to these empirical reasons for wishing to know more about the DIDS-binding proteins of non-erythroid cells, there is reason to think that the removal of lactate and the buffering of intracellular protons by $HCO_3^-$ transported from extracellular fluid ought to assume particular importance in malignant cells. The maintenance of intracellular pH depends, essentially, on three pathways: (i) the $Na^+/H^+$ (amiloride-sensitive) exchanger, (ii) the inorganic anion transporter and (iii) the organic anion transporter (Pace & Travin, 1983). It might be expected that anion-transport would be rapid in malignant cells because the high rate of glycolysis, which is characteristic of malignant cells, will result in the production of large amounts of lactic acid (Vaupel *et al.*, 1981). If a drop of intracellular pH is to be avoided, exchange of $Cl^-/HCO_3^-$, $Na^+/H^+$ and the efflux of lactate must be rapid (Roos & Boron, 1981). In addition anion transport may be an important modulator of cell growth and proliferation. It has recently been shown (L'Allemain *et al.*, 1984) that if the $HCO_3^-$ is removed from the growth medium of Chinese hamster lung fibroblasts then blockade of the $Na^+/H^+$ exchanger with amiloride prevents serum-stimulated initiation of growth. Since DIDS specifically inhibits the flux of inorganic anions at micromolar concentration (Cabantchik & Rothstein, 1974; Deuticke *et al.*, 1982) and at higher concentrations can also block lactate transport (Jennings & Adams-Lackey, 1982) it was chosen as the most appropriate probe to investigate the structure and regulation of the anion transporter of normal and malignant cells.

This paper describes the preparation of a monoclonal antibody (E8) against DIDS and describes the application of this antibody to study the amount and heterogeneity of expression of the DIDS-binding proteins of normal and tumorigenic human cells. This information is used to compare and contrast the DIDS-binding protein with the M/27 antigen.

### Materials and methods

### *Cell culture*

Cells were maintained in Dulbecco's Modified Eagle Medium (Gibco, No. 430-2100) in an incubator maintained at 5% (v/v) $CO_2$ in air at 37°C. The medium was supplemented with either 10% (v/v) foetal calf serum (FCS) or 5% foetal calf serum and 5% (v/v) newborn calf serum (NCS) together with 1 mM L-glutamine, 20 mM sodium pyruvate, 50 Iu ml$^{-1}$ penicillin G, 50 Iu ml$^{-1}$ streptomycin sulphate and 80 Iu ml$^{-1}$ neomycin sulphate. The cultures were checked at regular intervals for mycoplasma by the method of Chen (1977). Cells

Received 26 July 1984; accepted 6 September 1984.

© The Macmillan Press Ltd., 1984

to be harvested for the analysis of DIDS-binding proteins were detached from the glass bottles with 7 mM $Na_2HPO_4$-$2H_2O$, 3 mM $NaH_2PO_4$-$2H_2O$, 137 mM NaCl (PBS) containing 0.02% (w/v) EDTA. A suspension culture of HeLa cells was grown in Minimal Essential Medium modified for suspension culture (S-MEM, Gibco No. 410-1800). pH was maintained with 5% (v/v) $CO_2$ in air and 25 mM $NaHCO_3$. The medium was supplemented as described above using 5% (v/v) FCS/5% (v/v) NCS.

*Conjugation of DIDS to proteins*

DIDS (Pierce Chemical Company, No. 20047) was covalently attached to proteins and unbound DIDS removed by dialysis. The protein solutions were prepared at 1–10 mg ml$^{-1}$ in PBS and DIDS was added to a 10–100 molar excess. The solution was mixed for 1.5–4 h at 20°C, in the dark, and the reaction continued overnight at 4°C. The solution was then extensively dialyzed against PBS and stored at −20°C. Proteins used were: bovine serum albumin, BSA (Sigma No. A7638), ovalbumin (Sigma No. A5503), human gammaglobulin, HGG (Sigma No. G2011) and carbonic anhydrase, CA (Sigma No. C7500).

*Preparation of monoclonal anti-DIDS antibodies*

Female BALB/c mice were initially immunized subcutaneously with 50 μg DIDS-HGG in Freund's complete adjuvant. The immunization was repeated 3 times over 3 months and a final intraperitoneal injection of 200 μg of the antigen was given 4 days before removing the spleen for fusion. The non-secreting myeloma NS-1 was used for fusion and standard techniques were followed (Köhler & Milstein, 1975).

*Solid-phase radioimmunoassay*

The presence of antibodies against DIDS was measured by radio-immunoassay of culture supernatants on Immunolon 2 Removawell strips (Dynatech Laboratories, No. 011-010-6302) which were coated with a DIDS-ovalbumin conjugate at 15 μgml$^{-1}$ in PBS for 1 h at 37°C. BSA (5%, w/v) was used to block the remaining binding sites. Primary and secondary antibodies were incubated in the well for 1 h each and free antibody removed by washing with PBS. Ovalbumin was used as a negative control.

In experiments to demonstrate the specificity of E8 for DIDS (Figure 1), the protein solutions were absorbed to the wells at 100 μg ml$^{-1}$ in PBS for 1 h at 37°C and blocked with BSA, as before, but thereafter additional washing steps were used. Following the BSA-blocking the wells were washed with 25 mM Tris/HCl pH 7.4, 140 mM NaCl (TBS) for 5 min then with TBS containing 0.05% (v/v) Triton × 100 for 10 min and finally washed in TBS again for 5 min. Fifty μl of the primary antibody solution at 25 g ml$^{-1}$ in TBS/1% FCS was applied per well. Unbound primary antibody was removed, after 1 h incubation at 20°C, by washing as before (2 min each step). [$^{125}$I]-rabbit antimouse antibody (RAM) was added at 2.6 × 10$^4$ cpm per 50 μl in TBS/1% FCS. After a further 1 h incubation the wash procedure was repeated (5, 10, 5 min) and the dry wells counted.

*The binding of DIDS to the cell membrane*

The concentration of DIDS necessary to saturate the high affinity binding sites was determined by a saturation-binding assay. Increasing concentrations of freshly prepared DIDS were added to 4 × 10$^6$ HEp-2 cells suspended in 5 ml of PBS. The reaction proceeded in the dark for 10 min at 4°C with frequent mixing. The reaction was stopped by adding an equal volume of TBS containing 5 mM 1,2-diamino ethane (Fluka, K3596-/25/2/4). The cells were pelleted at 400*g* for 3 min and resuspended in 5 ml of TBS. The cells were pelleted again and resuspended to 2 × 10$^7$ cells ml$^{-1}$ in PBS. The amount of DIDS bound to the cell membrane was determined by saturation-binding assay.

*Saturation- and Trace-binding assays*

The basic method of Letarte-Muirhead *et al.*, (1975) was followed. One hundred μl of E8 culture supernantant or 50 μl of purified E8 at 25 mg ml$^{-1}$ was used for the primary antibody incubation. The [$^{125}$I]labelled rabbit anti-mouse IgG (H + L) (Miles No. 65.157) was used at 25 μg ml$^{-1}$ or higher. Cells were resuspended to 2 × 10$^7$ ml$^{-1}$ in PBS and 50 μl was used per well. Determinations were made in triplicate or quadruplicate.

*Flow cytometry*

HeLa spinner cells were washed 4× in PBS and DIDS conjugated to the membrane as described previously. Two hundred and fifty μl of cells at 2 × 10$^7$ cells ml$^{-1}$ were resuspended in saturating amounts of ascites (M/27) or purified antibody (E8) prepared in TBS/1% FCS/0.02% $NaN_3$. (TBS/FCS/Az). Following an incubation of 1 h, the cells were washed with TBS/FCS/Az and incubated with 1:40 dilution of FITC labelled RAM (Dako, No. F232) for a further hour. The cells were washed 3 times and resuspended to 1 ml in PBS for analysis on the FACS (Beckton Dickinson, FACS IV). The cells were maintained at 4°C until the time of analysis.

### Results

### *Characterization of the monoclonal antibody*

The specificity of the E8 monoclonal antibody was determined by comparing the binding of E8 to four different proteins to which DIDS had or had not been conjugated. DIDS was mixed at 100:1 molar ratio (DIDS:protein) to samples of ovalbumin, bovine serum albumin, human gamma globulin and carbonic anhydrase as described in materials and methods. Another monoclonal antibody, JCS-2, was used as a negative control. JCS-2 is an IgG2a monoclonal anti-glycophorin (unpublished results). In all cases the binding of E8 to the unconjugated proteins was similar to the binding of the negative control and significantly lower than the binding of E8 to the DIDS-conjugated proteins (Figure 1). Hence, within the scope of the comparison E8 is specific for DIDS. The epitope or immunodominant region of DIDS is unknown but E8 is not absorbed by any of a panel of sulphated compounds including chondroitin 4-sulphate, heparin, fucoidon and carrageenan.

The E8 monoclonal antibody was shown to be of the IgG1 isotype by double diffusion against monospecific commercial antisera (data not shown).

**Figure 1** The specificity of binding of E8 monoclonal antibody for DIDS. Five $\mu$g of protein ($\pm$DIDS) were added per well to plastic microtitre dishes and the binding of E8, (□) and JCS-2, (▨) was measured by solid-phase radioimmunoassay. The bars represent 1 s.d. The assay was performed in quadruplicate.

### *The kinetics of binding of DIDS for the cell membrane*

The rate of binding of DIDS to HeLa cells followed biphasic kinetics, a rapid initial phase, which was completed in 5–10 min, was followed by a phase with a slower rate which did not reach saturation during the 60 min of the time-course studied. A separate experiment of the kinetics of the 0–10 min period confirmed the rapid initial binding of DIDS (unpublished observations). The biphasic rate of binding observed in these experiments is very similar to the binding of $H_2$ DIDS to the erythrocyte membrane at 20°C (Lepke *et al.*, 1976, Figure 3).

A measure of the affinity of binding of DIDS for its receptor was examined in HEp-2 cells (a human laryngeal carcinoma cell line). A concentration-dependent saturation was reached at 50–100 M with half-saturation at 16 M (Figure 2). These experiments measure only the rapid phase of binding since the reaction was terminated at 10 min. The value of 16 $\mu$M is intermediate between the Ki of DIDS for the inorganic anion transporter of the human erythrocyte, (Cabantchik & Rothstein, 1974; Lepke *et al.*, 1976) and the Ki of DIDS for the organic anion transporter which Jennings & Adams-Lackay (1982) found to be inhibited by 80% at 100 $\mu$M $H_2$ DIDS. It is reasonable to expect that non-erythroid cells have transporters for both inorganic and organic anions (Spencer & Lehringer, 1976) but no systematic comparisons are available in the literature to suggest the relative abundance of each.

**Figure 2** The kinetics of binding of DIDS to the cell membrane. An increasing concetration of DIDS was added to a constant number of HEp2 cells at 4°C for 10 min and the resulting amount of DIDS bound to the membrane was measured by a saturation-binding assay. *Insert*: Time-course of binding of 100 $\mu$M DIDS to HeLa cells at 4°C. The bars represent 1 s.d. Each point was determined in triplicate.

*A comparison of the expression of DIDS-binding protein on normal and tumorigenic cells.*

A number of different cell types were conjugated with DIDS (at saturation) and the amount of DIDS bound 10%[6] cells was measured by the saturation-binding assay. Initial experiments showed that HEp-2 had significantly ($P<0.001$) more DIDS-binding sites than the non-tumorigenic MRC-5 (a human foetal fibroblast) cells. A more stringent comparison was then made. The number of DIDS-binding sites was compared on a series of hybrids between D98/AH-2 (a HeLa variant deficient in HGPRT) and normal human fibroblasts (Stanbridge *et al.*, 1982). No difference was found in the number of DIDS-binding sites between the tumorigenic variants (5E and 39E) and those in which tumorigenicity had been suppressed (5L and ESH) and (Figure 3).

**Figure 3** A compairson of the number of DIDS-binding sites on normal, malignant and hybrid cell lines. The bar represents one standard deviation. Tumorigenic cell lines unmarked; non-tumorigenic cell lines cross-hatched. Each point was determined in quadruplicate.

*A comparison of the expression of the DIDS-binding protein and the M/27 determinant*

Previous experiments with M/27 monoclonal antibody have demonstrated the antigen to be expressed heterogeneously; a small proportion of the cell population is labelled much more heavily than the bulk of the population (Gingrich *et al.*, 1981*b*; Banyard & White, 1984). The cells heavily labelled with M/27 monoclonal antibody have two features in common, they are in S-phase of the cell cycle (Gingrich *et al.*, 1981*b*) and they transport glucose more rapidly than the cell population as a whole (Banyard & White, 1984). Because the binding of DIDS to the cell-surface increases the number of M/27 binding sites it was suggested that the DIDS-binding protein and the M/27 antigen must either be the same, or be clearly associated in the cell membrane (Banyard *et al.*, 1982). But since the binding of DIDS does not compete with M/27 (Banyard *et al.*, 1982) the binding sites cannot be identical nor within the molecular radii of M/27 plus DIDS. They could occur at distant sites on the same molecule; in which case binding of DIDS could exert an allosteric effect at a distant site of the same molecule which facilitates the binding of M/27 to its epitope.

The fluorescence activated cell sorter (FACS) was used to distinguish between the two possibilities (i) that the binding sites occur on the same molecule, in which case their expression should be co-regulated or (ii) that the binding sites occur on different molecules so that non-coordinate regulation might occur. The heterogeneity of binding of M/27 is recognised on the FACS as a skewing of the fluorescence distribution to the right. If all cells within the population bind the monoclonal equally, a single peak, which follows a Poisson distribution, should be recorded. Figure 4 shows E8 labels a single peak of cells homogeneously whereas the cells labelled with M/27 antibody demonstrate a skewed distribution of more fluorescent cells. The proportion of cells in the skewed tail (more fluorescent than channel 59) is ~20% of the total cells counted, a figure in agreement with previous work (Gingrich *et al.*, 1981*b*; Banyard & White, 1984). The absolute number depends, of course, on the arbitrary point of cut-off which was, in this instance, taken as the point of divergence between the E8 and the M/27 profile (Figure 4). The value of 20% in the skewed tail was determined by subtracting the percent of

**Figure 4** A FACS analysis of the fluorescence heterogeneity, A, and size heterogeneity, B, of HeLa spinner cells conjugated with 100 $\mu$M DIDS and labelled with either E8 monoclonal antibody, profile 1, or M/27 monoclonal antibody, profile 2. Both profiles were aligned so the peaks (determined by the instrument) coincided to channel 31/32. The skewing is illustrated by the arrow.

cells greater than channel 59 for E8 from the percent greater than channel 59 for M/27. These results suggest that the DIDS-binding is not coordinately expressed with the M/27 determinant and is therefore presumably on a separate molecule. The dissimilar number of binding sites for M/27 and E8 on HeLa spinner cells support this conclusion. HeLa spinner cells have nearly 4 times more DIDS-sites than M/27 binding sites (Table I).

**Table 1** A comparison of the number of DIDS- and M/27-binding sites on HeLa cells.

| *Antibody* | *Antibody bound (CMP/10⁶ cells)* | *Number* | *Significance* |
|---|---|---|---|
| M/27 | 60,676 ± 6284 | 4 | — |
| E8[a] | 237,428 ± 25616 | 4 | $P<0.001$ |

[a]DIDS was bound to cells at 100 $\mu$M, for 10 min and a saturation binding assay done to determine the amount of antibody bound. Specific activity of [$^{125}$I]-RAM was $4.5\times10^5$ cpm $\mu g^{-1}$. Results are expressed + s.d.

### Discussion

The concentration at which DIDS half-saturates HEp-2 cells was found to be 16 $\mu$M, a value which is intermediate between the Ki of DIDS for the inorganic anion transporter (Band 3) of the human erythrocyte, which is about 1 $\mu$M (Cabantchik & Rothstein, 1974; Lepke *et al.*, 1976) and the Ki of DIDS for the organic anion transporter which Jennings & Adams-Lackey (1982) found to be inhibited by 80% at 100 $\mu$M $H_2$ DIDS. It should be noted that the DIDS and $H_2$ DIDS have slightly different requirements for binding to their substrates (Lepke *et al.*, 1976) but both effectively inhibit inorganic anion transport. The apparently simple kinetics of binding over 5–10 min suggests a single major binding site but the use of an indirect-antibody assay to determine this point in detail is inappropriate because of the complex kinetics which characterise the antigen-antibody reaction. Since the apparent Ki is intermediate between the inorganic and the organic anion transporter no rational guess can be made about which one is the major DIDS-binding protein of malignant cells but the molecules appear to be structurally distinct. The lactate transporter of the rabbit erythrocyte membrane labelled by [$^3$H]$H_2$-DIDS has a relative molecular mass ($M_r$) on SDS-PAGE of $4.3\times10^4$ (Jennings & Adams-Lackey, 1982) whereas the inorganic anion transporter has a relative molecular mass of $9.5\times10^4$ (Cabantchik & Rothstein, 1974).

The data comparing the number of DIDS-binding sites on tumorigenic and non-tumorigenic cells suggests that the $V_{max}$ for lactate and inorganic anions is not rate-limiting in tumorigenic cells. This finding supports the work of Johnson *et al.*, (1980) which shows that lactate transport had to be almost completely blocked before intracellular pH fell. Spencer & Lehninger (1976) also found that the maximal rate of lactate transport was far in excess of the production of lactate by glycolysis in Ehrlich ascites tumour cells.

Three independent pieces of information suggest that the DIDS-binding protein and the M/27 antigen are different molecules. Firstly, the DIDS-binding protein, measured indirectly with the E8 monoclonal antibody, is expressed homogeneously on all cells within the population whereas M/27 is not. Secondly, DIDS does not compete with M/27 but in fact enhances binding of M/27 (Banyard *et al.*, 1982). This observation suggests that when the DIDS-binding protein and M/27 antigen coexist in the same cell there is a close topological association between them. The importance of such membrane complexes is supported by the observation that DIDS and ouabain both independently cause an alteration of the NMR-resonance of [$^{31}$P]-2,3-diphosphoglycerate through the membrane (Fossel & Solomon, 1983) suggesting that long-range allosteric effects may operate between apparently unrelated membrane molecules. Finally, the dissimilar levels of expression of E8 and M/27 on the same cell also supports the idea that the proteins are distinct entities. A direct comparison of the number of sites per cell should not however be overinterpreted since the stoichiometry of [$^{125}$I]-RAM binding to an IgM monoclonal antibody (M/27) and to a $IgG_1$ monoclonal antibody (E8) is likely to be different. Furthermore since the M/27 determinant is heterogenously expressed the number of sites per cell can only be expressed as an arithmetic mean of the binding to the whole population.

The possible topological assocation between the M/27 antigen and the DIDS-binding protein is currently being studied directly by the purification of the DIDS-binding component. Previous, extensive attempts to purify the M/27 determinant in quantity have been unsuccessful. It is hoped that this new approach will finally resolve the relationship between the DIDS-binding protein and the Bramwell-Harris $1\times10^5$ $M_r$ glycoprotein (Bramwell & Harris, 1978).

The technical help of Miss R. Stokes is gratefully acknowledged. The help of Mrs K. Rabl in typing the manuscript is also gratefully acknowledged. I thank Dr. R.B. Ashman and Mr. D. Light for their help with the FACS analysis.

**References**

BANYARD, M.R.C., BRAMWELL, M.E., RODGERS, A. & WHITE, M.K. (1982). Anion and Glucose transport in malignant and non-malignant cells. In: *Membranes in Tumour Growth.* (Eds. Galeotti, *et al.,*) Elsevier Biomedical Press, Amsterdam p. 503.

BANYARD, M.R.C. & WHITE, M.K. (1984). Association of an integral membrane protein with glucose transport and with anion transport. *J. Cell Sci.,* **67,** 45.

BRAMWELL, M.E. & HARRIS, H. (1978). Some further information about the abnormal membrane glycoprotein associated with malignancy. *Proc. R. Soc. Lond. B.,* **203,** 93.

CABANTCHIK, Z.I. & ROTHSTEIN, A. (1974). Membrane proteins related to anion permeability of human red blood cells. *J. Memb. Biol.,* **15,** 207.

CHEN, T.R. (1977). In situ detection of mycoplasma contamination in cell cultures by fluorescent Hoechst 332-58 stain. *Exp. Cell Res.,* **104,** 255.

DEUTICKE, B., BEYER, E. & FORST, B. (1982). Discrimination of three parallel pathways of lactate transport in the human erthrocyte membrane by inhibitors and kinetic properties. *Biochim. Biophys. Acta.,* **684,** 96.

FOSSEL, E.T. & SOLOMON, A.K. (1983). Relation between red cell membrane Na, K-ATPase and Band 3. In: *Curr. Top. Membranes Trans.,* **19,** 481.

GINGRICH, R.D., WOUTERS, M., BRAMWELL, M.E. & HARRIS H. (1981*a*). Immunological delineation in normal and malignant cells of a membrane protein involved in glucose transport. I. Preparation and properties of the antibody. *J. Cell Sci.,* **52,** 99.

GINGRICH, R.D., WOUTERS, M., BRAMWELL, M.E. & HARRIS, H. (1981*b*). Immunological delineation in normal and malignant cells of membrane protein involved in lucose transport. II. Function of the antogen. *J. Cell Sci.,* **52,** 121.

JENNINGS, M.L. & ADAMS-LACKEY, M. (1982). The rabbit erythrocyte membrane protein associated with L-lactate transport. *J. Biol. Chem.,* **257,** 12866.

JOHNSON, J.H., BELT, J.A., DUBINSKI, W.P. ZIMNIAK, A. & RACKER, E. (1980). Inhibition of lactate transport in Ehrlich ascites tumour cells and human erythrocytes by a synthetic anhydride of lactic acid. *Biochimie,* **19,** 3836.

KÖHLER, G. & MILSTEIN. C. (1975). Continuous cultures of fused cells secreting antibody of a predefined specificity. *Nature,* **256.** 459.

L'ALLEMAIN, G., FRANCHI, A., CRAGOE, E. & POUYSSEGUR, J. (1984). Blockade of the $Na^+/H^+$ antiport abolishes growth factor-induced DNA synthesis in fibroblasts. *J. Biol. Chem.,* **259,** 4313.

LEPKE, S., FASOLD, H., PRING, M. & PASSOW, H. (1976). A study of the relationship between inhibition of anion exchange and binding to the red blood cell membrane of 4,4′-diisothiocyanostilbene-2,2′-disulfonic acid (DIDS) and its dihydro derivative ($H_2$ DIDS). *J. Memb. Biol.,* **29,** 147.

LETARTE-MUIRHEAD, M., BARCLAY, A.N. & WILLIAMS, A.F. (1975). Purification of the Thy-1 molecule, a major cell-surface glycoprotein of rat thymocytes. *Biochem. J.,* **151,** 685.

PACE, C.S. & TARVIN, J.T. (1983). pH modulation of glucose-induced electrical activity in B-cells: Involvement of Na/H and $HCO_3$/Cl antiporters. *J. Memb. Biol.,* **73,** 39.

ROOS, A. & BORON, W.F. (1981). Intracellular pH. *Physiol. Rev.,* **61,** 296.

SPENCER, T.L. & LEHNINGER, A.L. (1976). L-lactate transport in Ehrlich ascites-tumour cells. *Biochem. J.,* **154,** 405.

STANBRIDGE, E.J., DER, C.J. & DOERSEN, C-J., & 4 others (1982). Human cell hybrids: Analysis of transformation and tumorigenicity. *Science,* **215,** 252.

VAUPEL, P.W., FRINAK, S. & BICHER, H.I. (1981). Heterogenous oxygen partial pressure and pH distribution in C3H mouse mammary adenocarcinoma. *Cancer Res.,* **41,** 2008.