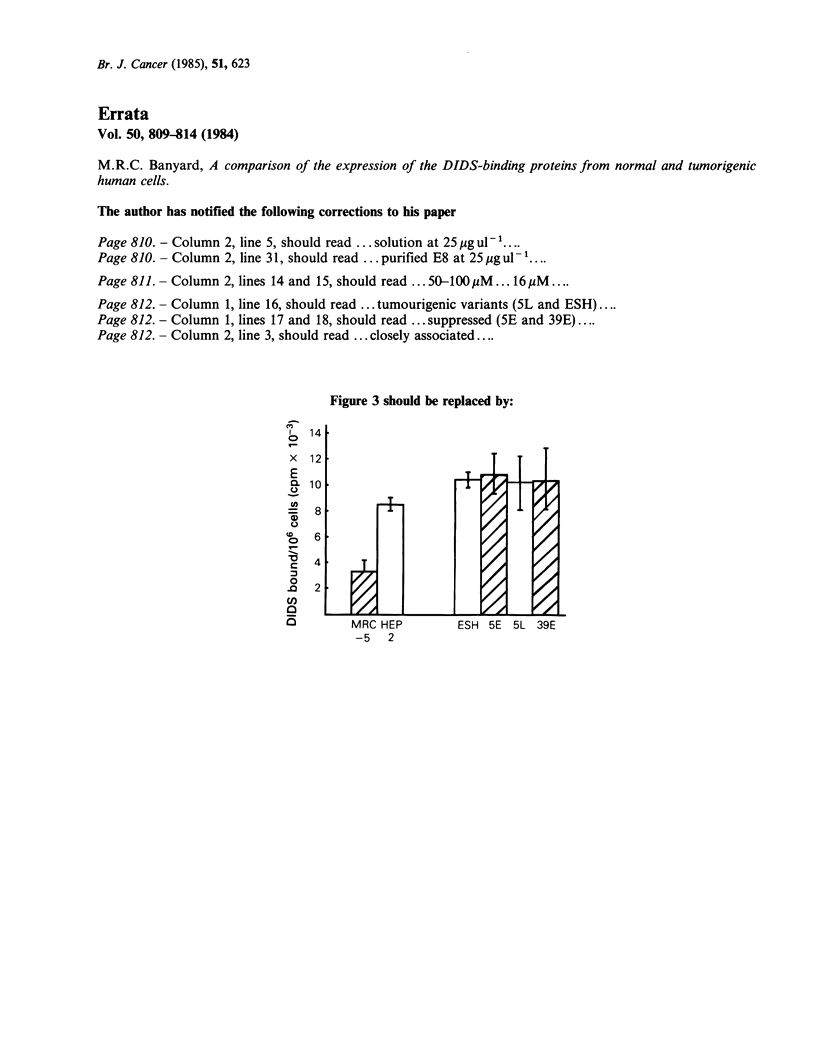# Erratum

**Published:** 1985-04

**Authors:** 


*Br. J. Cancer* (1985), **51**, 623

### Errata

### Vol. 50, 809–814 (1984)

M.R.C. Banyard, *A comparison of the expression of the DIDS-binding proteins from normal and tumorigenic human cells.*

**The author has notified the following corrections to his paper**

*Page 810.* – Column 2, line 5, should read ... solution at 25 $\mu$g ul$^{-1}$....
*Page 810.* – Column 2, line 31, should read ... purified E8 at 25 $\mu$g ul$^{-1}$....

*Page 811.* – Column 2, lines 14 and 15, should read ... 50–100 $\mu$M ... 16 $\mu$M....

*Page 812.* – Column 1, line 16, should read ... tumourigenic variants (5L and ESH)....
*Page 812.* – Column 1, lines 17 and 18, should read ... suppressed (5E and 39E)....
*Page 812.* – Column 2, line 3, should read ... closely associated....

**Figure 3 should be replaced by:**